# A Case with Multiple Pathologies in the Pancreatic Head

**DOI:** 10.3390/biomedicines12081762

**Published:** 2024-08-05

**Authors:** Miroslav Vujasinovic, Sam Ghazi, Nikolaos Kartalis, Maria Gustafsson Liljefors, Melroy A. D’Souza, Poya Ghorbani, J.-Matthias Löhr

**Affiliations:** 1 Department of Upper Abdominal Diseases, Karolinska University Hospital, 14186 Stockholm, Sweden; maria.gustafsson-liljefors@regionstockholm.se (M.G.L.); melroy.dsouza@regionstockholm.se (M.A.D.); poya.ghorbani@regionstockholm.se (P.G.); matthias.lohr@ki.se (J.-M.L.); 2 Department of Medicine Huddinge, Karolinska Institutet, 14186 Stockholm, Sweden; 3 Department of Pathology and Cancer Diagnostics, Karolinska University Hospital, 14186 Stockholm, Sweden; sam.ghazi@regionstockholm.se; 4 Department of Clinical Science, Intervention and Technology, Karolinska Institutet, 14186 Stockholm, Sweden; nikolaos.kartalis@regionstockholm.se; 5 Department of Radiology, Karolinska University Hospital, 14186 Stockholm, Sweden

**Keywords:** autoimmune pancreatitis, paraduodenal pancreatitis, IPMN, pancreatic ductal adenocarcinoma, follicular pancreatitis

## Abstract

**Objectives:** Autoimmune pancreatitis (AIP) type 1, paraduodenal (groove) pancreatitis, and follicular pancreatitis are rare clinical entities whose diagnosis may be challenging, given the potential imaging overlap with pancreatic cancer. **Methods:** We performed a retrospective analysis of the medical chart of a patient with multiple pancreas pathologies. **Results:** We present a case with multiple pancreas pathologies, including a poorly differentiated ductal adenocarcinoma of pancreatobiliary type, an intraductal papillary mucinous lesion (pre-existing lesion of IPMN type), and an inflammatory process with complex features, in which paraduodenal (groove) pancreatitis, follicular pancreatitis, and IgG4-related pancreatitis (AIP type 1) were also present. **Conclusions:** The diagnosis of AIP and paraduodenal pancreatitis is not always straightforward, and in some cases, it is not easy to differentiate them from pancreatic cancer. Surgery should be considered in patients when a suspicion of malignant/premalignant lesions cannot be excluded after a complete diagnostic work-up.

## 1. Introduction

IgG4-related disease (IgG4-RD) is a systemic autoimmune disease defined by elevated levels of IgG4 in serum and affected tissues [1]. The term autoimmune pancreatitis (AIP) was coined in the 1990s [2] and is classified into type 1 (IgG4-RD) and type 2 (mostly related to inflammatory bowel disease) [3]. IgG4-RD in the pancreas (type 1 AIP) is associated with lymphoplasmacytic sclerosing pancreatitis as the histological feature, increased serum levels of IgG4 in most patients, and IgG4+ cells in the affected tissues [1,4].

Paraduodenal (groove) pancreatitis is a specific form of chronic pancreatitis (CP) occurring in and around the duodenal wall [5,6]. Both AIP type 1 and paraduodenal pancreatitis may represent a diagnostic challenge, given their potential imaging overlap with pancreatic cancer [4,6].

Follicular pancreatitis is a very rare type of focal CP and is often mistaken for pancreatic neoplasms, histologically characterized by extensive lymphoid follicular formation with reactive germinal centers [7].

Intraductal papillary mucinous neoplasm (IPMN) of the pancreas is characterized by the dilatation of the main pancreatic duct and/or its side branches and is a precursor of pancreatic ductal adenocarcinoma (PDAC) [8].

We are presenting a rare case of a patient with multiple pathologies in the pancreatic head.

## 2. Methods

We performed a retrospective analysis of the medical chart of a patient, based on approval from the Clinic Ethical Committee in Stockholm (EPN Dnr. 2016/1571-31; Dnr. 2020-02209; Dnr. 2016/2542-31/1; Dnr. 2019-03345). The patient whose case is described in this manuscript has been informed and has fully agreed to publication in an anonymous manner, and her approval has been documented in electronic medical charts.

## 3. Case Report

**Figure 1:**
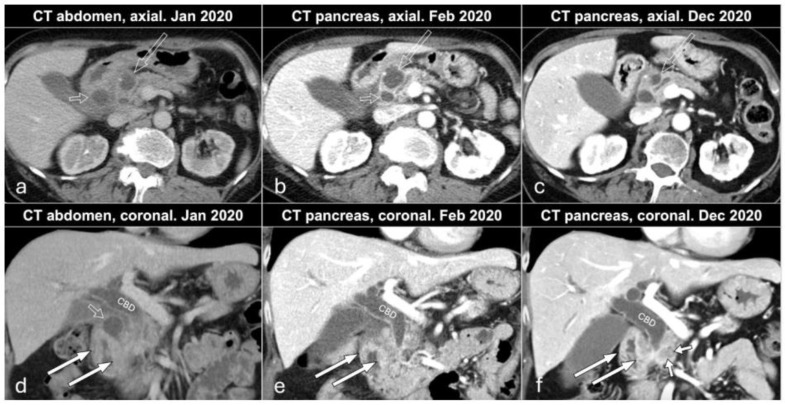
Axial (**a**–**c**) and coronal (**d**–**f**) contrast-enhanced images of CT abdomen in January (**a**,**d**) and CT pancreas in February (**b**,**e**) and December (**c**,**f**) 2020. CBD: common bile duct; open white arrows: cystic lesion in the pancreatic head; open short white arrows: cystic lesion in the pancreatic groove; white arrows: wall thickening of the descending portion of duodenum; short white arrows: pancreatic cancer.

A 68-year-old female presented at a primary care outpatient clinic in January 2020 due to abdominal pain, weight loss (11 kg in total in one year), and frequent (7–9 per day) watery stools (sometimes fatty/oily stools). The patient had a 45-year history of smoking (approximately 15 pack-years of smoking) and very rarely drank alcohol. She had a 20-year history of kidney stones, and her abdominal pain disappeared after treatment with non-steroid anti-inflammatory drugs (NSAIDs) and tramadol. The patient was clinically diagnosed as suffering pain related to kidney stones, which was confirmed with computed tomography (CT) of the kidneys showing five stones < 5 mm in the right kidney and three stones < 5 mm in the left kidney. Routine laboratory results showed slightly elevated liver function tests (aspartate aminotransferase 0.7 μkat/L, alanine transaminase 1.05 μkat/L, and alkaline phosphatase 2.2 μkat/L), and CT of the abdomen and pelvis was performed (Figure 1a,d). In summary, there were inflammatory changes in the head of the pancreas, hepatoduodenal ligament, and peripancreatic fat, wall thickening of the second part of the duodenum, presence of cystic lesions in the pancreatic groove, and head and regional lymphadenopathy. Additionally, there was dilatation of the common bile duct (1.6 cm) and mild dilatation of the main pancreatic duct in the body and tail of the pancreas (5 mm) (Figure 1a,d). Gastroscopy in January 2020 showed mild bleeding of the edematous and vulnerable mucosa in duodenal pars descendens. The endoscopist described in the report that in this part of the duodenum the passage was somehow difficult and the expansion of the duodenal lumen after CO2 insufflation was not fully successful. There were no signs of ulcerations or tumor. Biopsy was performed, and the histopathology showed normal mucosa and Brunner glands. The patient was referred to a surgeon (February 2020), who started treatment with esomeprazole (due to the endoscopic findings) and pancreatic enzyme replacement therapy (PERT) (due to the history of weight loss and steatorrhea). The case was presented and discussed at the multidisciplinary team (MDT) pancreas conference at our hospital, and the team recommended CT of the pancreas and endoscopic ultrasound (EUS). CT of the pancreas (February 2020) confirmed the findings of inflammatory changes in the head of the pancreas, hepatoduodenal ligament, and peripancreatic fat, wall thickening of the second part of the duodenum (more prominent changes in the medial aspect of duodenum compared to the examination in January 2020), regional lymphadenopathy and dilatation of the common bile, and mild dilatation of the main pancreatic duct in the body and tail of the pancreas (Figure 1b,e). One of the cystic lesions in the head of the pancreas increased from 2 to 2.5 cm, whereas another cystic lesion in the pancreatic groove decreased from 2 to 1 cm.

The patient had a surgical consultation at the outpatient clinic (March 2020) and reported a marked improvement after PERT and esomeprazole treatment (she was pain-free and had gained 10 kg in weight). Control gastroscopy and EUS were recommended to ensure duodenal mucosa healing and to confirm pancreas morphology seen by CT, but the patient decided to wait before undergoing the procedures because of her clinical improvement and her wish to avoid hospital visits during the COVID-19 pandemic. However, endoscopic controls (gastroscopy and EUS) were performed in October 2020. Gastroscopy showed normal mucosa in the duodenum and other parts of the upper gastrointestinal system. Biopsy of the duodenum showed normal histopathological findings. EUS showed an atrophic and slightly inhomogeneous appearance of the pancreas parenchyma and 2.2 mm width of main pancreatic duct. A 2.5 × 1.6 cm thin-walled cyst was described in the head of the pancreas. No tumor was suspected, and no nodular changes were seen.

**Figure 2:**
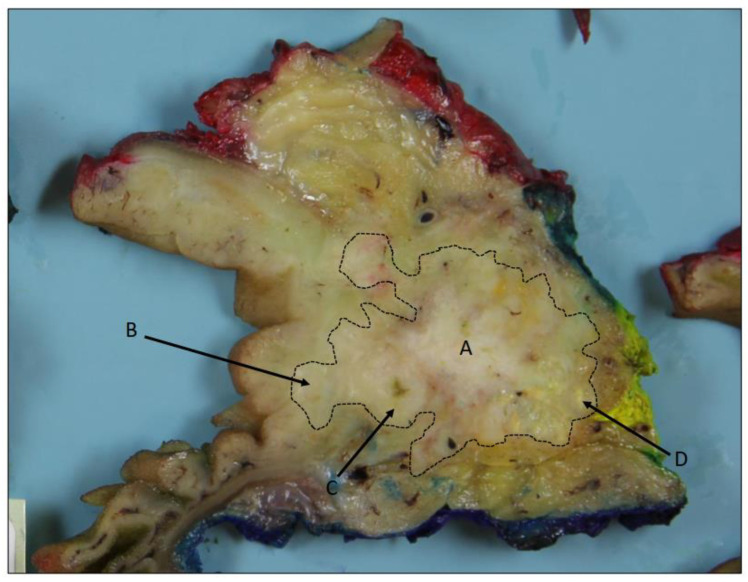
Axial slice showing a poorly circumscribed, solid, yellow-white tumor (A) in the pancreatic head. Suspected invasion of the duodenal wall (B), common bile duct (C), and peripancreatic fat (D). Main pancreatic duct obliterated by tumor and not visible.

**Figure 3:**
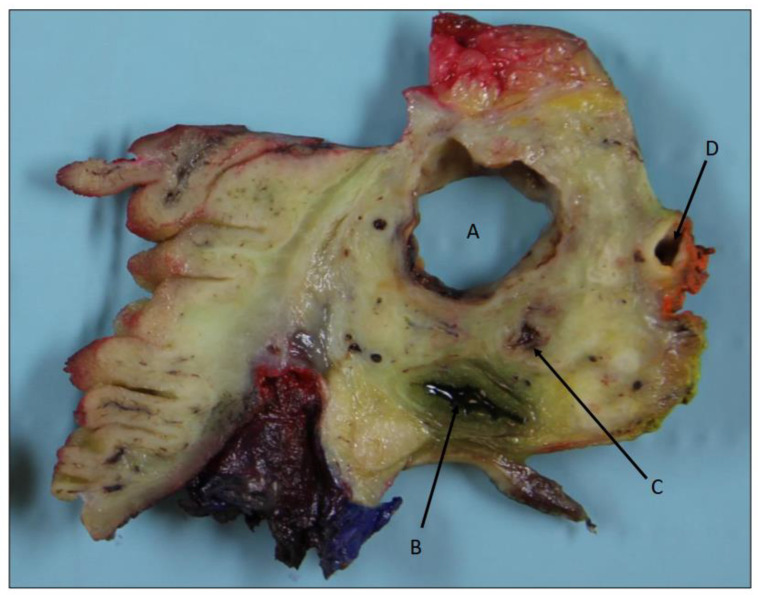
Axial slice of cranial caput showing a non-mucinous cyst (A) close to the anterior pancreatoduodenal groove and duodenal wall. No papillary structures or communication with the ductal system. Common bile duct (B), main pancreatic duct (C), and resected part of superior mesenteric vein (D) are also visible.

In December 2020, the patient came for a clinical check-up at the pancreatic outpatient clinic. Clinical examination showed jaundice, and laboratory tests showed elevated liver function tests (aspartate aminotransferase 4.06 μkat/L, alanine transaminase 9.01 μkat/L, gamma-glutamyl transferase 40.4 kE/L, alkaline phosphatase 11.5 kE/L, bilirubin 89 μmol/L), CA 19-9 (199 kE/L), IgG4 (2.82 g/L), and CRP (9 mg/L). CT of the pancreas showed mild regress of the inflammatory changes in the area and of the cystic lesions in the pancreatic groove and head, as well as of the regional lymphadenopathy. Wall thickening of the medial aspect of the second part of the duodenum remained, and additionally, there was a newly appearing hypovascular 1.5 cm solid mass medially in the head of the pancreas, which caused a further dilatation of the common bile duct from 1.6 to 2 cm (whereas the main pancreatic duct in the body and tail of the pancreas remained unchanged at 5 mm) (Figure 1c,f). After discussion at an MDT conference, a Whipple’s procedure was performed. Gross examination of the specimen after axial slicing showed a poorly circumscribed 1.6 cm solid yellow-white tumor in the pancreatic head with suspected invasion of the peripancreatic fat, ampulla of Vater, common bile duct, duodenal wall, and resected part of the superior mesenteric vein (Figure 2). Cranial to the tumor, in the region of the anterior pancreatoduodenal groove, a 2.4 cm non-mucinous unilocular cyst with a fibrotic wall was found (Figure 3). No papillary structures or obvious communication with the duct system were seen. The surrounding pancreatic and peripancreatic tissue showed fibrosis, and in the adjacent duodenum, a diffuse thickening of the mucosa and submucosa was noted.

**Figure 4:**
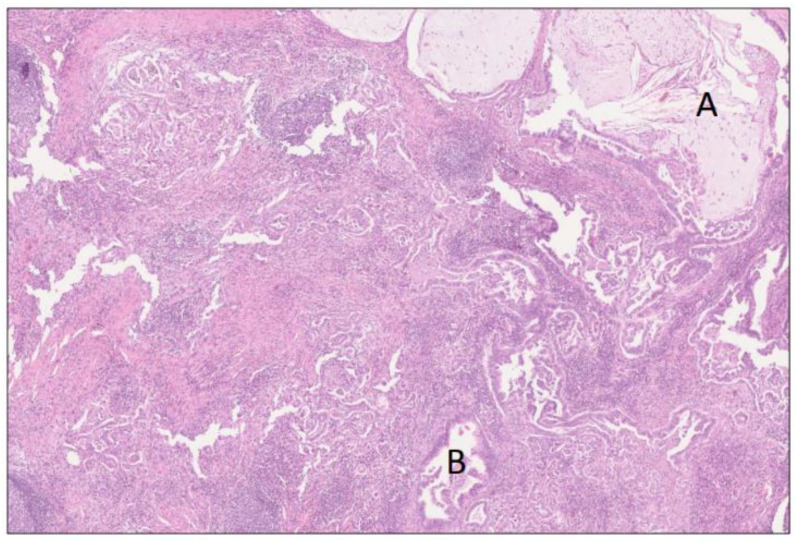
Poorly differentiated ductal adenocarcinoma of pancreatobiliary type showing areas of mucin production and large duct pattern (A), as well as cystic papillary pattern (B). H&E staining, 4×. The findings correspond to the tumor area (A) in Figure 2.

**Figure 5:**
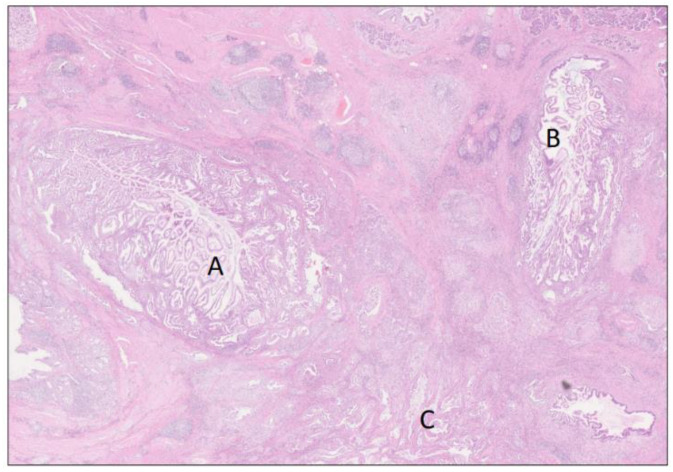
Intraductal papillary mucinous lesion of mixed gastric and intestinal type (immunohistochemistry not shown here) with high-grade dysplasia in main duct (A) and branch duct (B) adjacent to the invasive tumor (C). The lesion has the morphology of an IPMN, although the size (maximum diameter 6 mm) falls in between PanIN and IPMN. H&E staining, 1.5×. The findings correspond to the periphery of the tumor area (A) in Figure 2.

**Figure 6:**
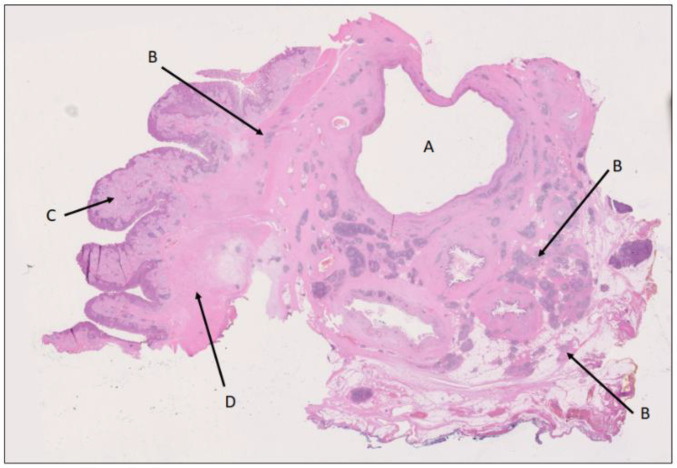
Large mount section from cranial caput showing the cyst in Figure 3 (A) surrounded by fibrosis and an abundance of lymphoid follicles (B) in the pancreatic head, peripancreatic fat, and duodenal wall. Note the hyperplasia of Brunner’s glands (C) and of the muscle layer of duodenum (D). H&E staining, 0.2×.

**Figure 7:**
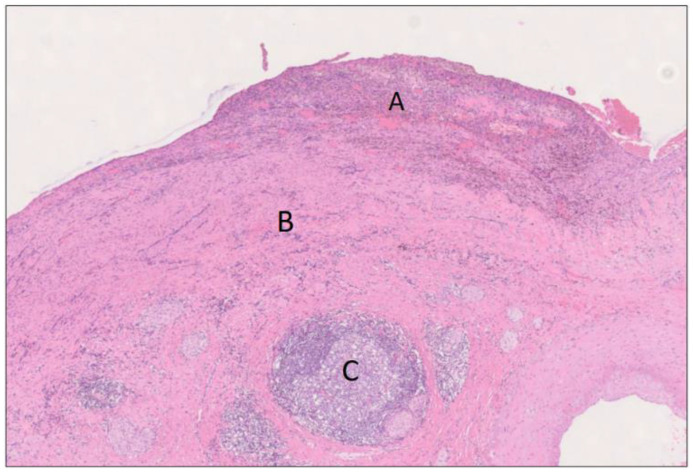
Higher magnification of the cyst wall in Figure 3 and Figure 6 showing granulation tissue (A) with lymphoplasmacytic inflammation, as well as foamy and hemosiderin-laden macrophages. No epithelial lining. In the periphery, fibrotic pancreatic tissue (B) with a lymphoid follicle (C). H&E staining, 2.5×.

**Figure 8:**
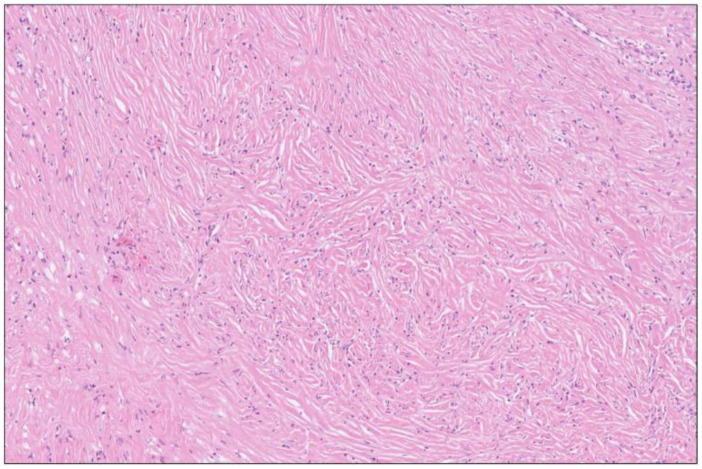
Storiform fibrosis of pancreatic parenchyma close to the cyst wall in Figure 3 and Figure 6 showing a cartwheel arrangement with radiating collagen fibers. H&E staining, 8×.

**Figure 9:**
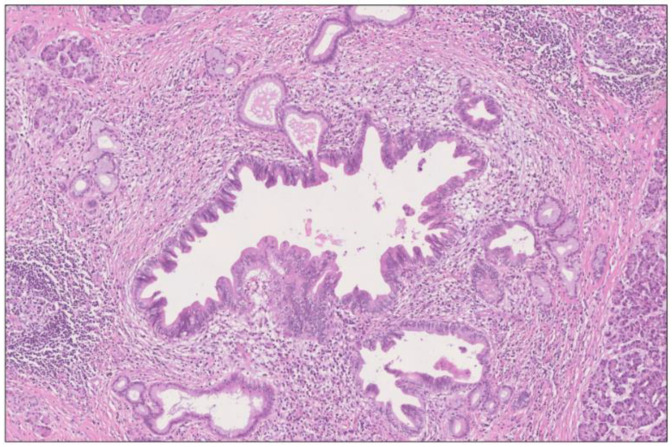
Periductal inflammation with a lymphoplasmacytic infiltrate surrounding a duct with neoplastic epithelium, probably representing duct cancerization. H&E staining, 8×. Findings correspond roughly to pancreatic tissue outside tumor area (A) in Figure 2.

**Figure 10:**
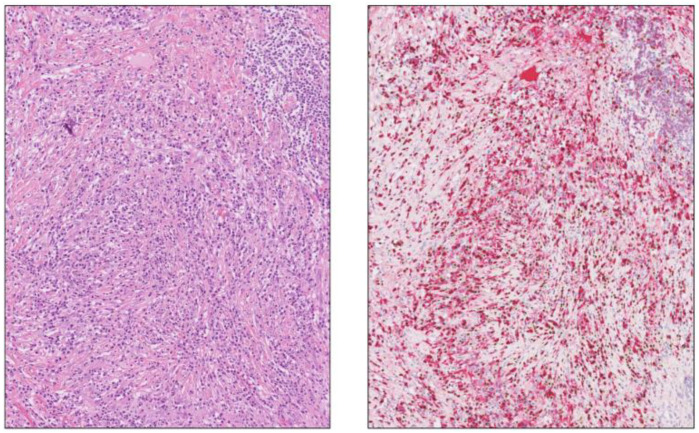
A dense lymphoplasmacytic infiltration in the non-tumorous pancreas in Figure 2 and Figure 3. Immunohistochemical double staining for MUM1 (brown)/IgG4 (red) shows focally >200 IgG4-positive plasma cells/HPFs with an estimated IgG4/IgG total ratio of 90%. H&E staining, 8×.

Microscopy from the tumor showed a poorly differentiated ductal adenocarcinoma of pancreatobiliary type with partial mucin production, as well as large duct and cystic papillary patterns (Figure 4). The infiltration of all adjacent structures above except the superior mesenteric vein was confirmed, together with metastases to two out of twenty-one regional lymph nodes. The findings corresponded to TNM stage pT2 N1, L1 V1 Pn1, and R1 because the distance to the margins facing the superior mesenteric vein and artery was less than 1 mm (eighth edition). In close relation to the invasive tumor, as well as further away, an intraductal papillary mucinous lesion with high-grade features and goblet cells was found in both the main duct and branch ducts (Figure 5). Positive immunostaining for CA19-9, CDX2, MUC2, MUC5AC, and MUC6 (not shown in figure) confirmed an epithelium of mixed gastric foveolar and intestinal type. This likely represented a pre-existing lesion of IPMN type, although the maximum duct diameter (6 mm) falls in between PanIN and IPMN. In addition, the foci of duct cancerization were detected. Histology from the cystic lesion showed pseudocyst-like features with a wall of fibrotic granulation tissue devoid of epithelial lining but with a lymphoplasmacytic infiltrate admixed with foamy and hemosiderin-laden macrophages (Figure 6 and Figure 7). The hyperplasia of Brunner’s glands, the thickening of the muscle layer, and fibrosis were seen in the nearby duodenal wall but no ectopic pancreatic tissue. Numerous enlarged lymphoid follicles with a germinal center and intact mantle zone were found throughout the pancreatic parenchyma, peripancreatic fat, and duodenal wall (Figure 6 and Figure 7). Immunohistochemistry confirmed reactive follicles with a normal distribution of B- and T-lymphocytes without clonality or other signs of lymphoma. The pancreatic head and peripancreatic tissue displayed a dense collagen fibrosis with a storiform pattern only focally, i.e., radiating fibers in a cartwheel fashion (Figure 8). A periductal lymphoplasmacytic infiltrate of varying density was seen in the pancreas and around the intrapancreatic bile duct and ampulla of Vater (Figure 9). This inflammatory reaction was more pronounced in the peritumoral compartment. The distribution was patchy, but the inflammation of the acinar parenchyma was hard to assess because of the severe acinar atrophy. No obliterative phlebitis or granulocytic epithelial lesions (GELs) were detected. Immunohistochemistry showed an average IgG4-positive plasma cell count of 80/high-power fields (HPFs) in hot spots, focally even up to 200/HPFs (Figure 10). The IgG4/IgG ratio (estimated with staining for MUM1) was 90% (cut-off value 50/HPFs and >40% for resection specimens).

In conclusion, besides a ductal adenocarcinoma with an intraductal IPMN-like lesion, the pancreatic head showed an inflammatory process with complex features where paraduodenal (groove) pancreatitis, follicular pancreatitis, or autoimmune pancreatitis (AIP) type 1 could be considered. The pancreatoduodenal groove cyst, together with the thickening of the duodenal wall and hyperplasia of Brunner’s glands, is in line with paraduodenal (groove) pancreatitis, although no ducts or ectopic pancreatic tissue were seen in the nearby duodenum. The numerous and prominent lymphoid follicles are consistent with follicular pancreatitis. No GELs, typical of AIP type 2, were detected. While the periductal lymphoplasmacytic infiltrate, parenchymal fibrosis, and elevated IgG4 plasma cell count are in line with AIP type 1, the picture is complicated by the peritumoral inflammatory reaction. Although no obliterative phlebitis and only a focal storiform pattern were present, the findings are suggestive of IgG4-related disease. The diagnosis of IgG4-related disease was confirmed with the elevated serum IgG4 = 8.54 g/L (reference values: 0.05–1.25 g/L). The postoperative phase was uneventful, and the patient started adjuvant chemotherapy (gemcitabine and capecitabine; eight applications) in February 2022. Unfortunately, a relapse was diagnosed in June 2022 (at the departure of the arteria hepatica communis, liver metastasis, and peritoneal carcinosis in the left flank), and palliative chemotherapy treatment (nab-paclitaxel and gemcitabine; five applications) started in July 2022. During 2023, the malignant disease was stable, but in January 2024, there was disease progress with further development of liver metastases. The patient is undergoing palliative chemotherapy at the time of writing this report.

## 4. Discussion

We present a case with multiple pathologies in the pancreatic head, including a poorly differentiated ductal adenocarcinoma of pancreatobiliary type, an intraductal papillary mucinous lesion (pre-existing lesion of IPMN type), and a complex inflammatory process where features of paraduodenal (groove) pancreatitis, follicular pancreatitis, and IgG4-related pancreatitis (AIP type 1) were also present. To the best of our knowledge, this is the first case with such a complex pancreas pathology.

Paraduodenal (groove) pancreatitis is a specific form of CP occurring in and around the duodenal wall (into the ‘groove’ area between the loop of the duodenum, the head of the pancreas, and the common bile duct) [5]. A recently published systematic review included 44 studies involving reports on 1404 patients with paraduodenal pancreatitis (mean age of 49 years, 86% males) [6]. Most of the patients (79%) had a history of excessive alcohol consumption and smoking (83%) [6], which is in line with our previously published experience on 35 patients (86% males, alcohol over-consumption in 81% and smoking in 90%) [5]. Paraduodenal pancreatitis represents a diagnostic challenge, given its potential imaging overlap with pancreatic cancer [9]. A systematic review on the imaging of paraduodenal pancreatitis identified 14 studies (CT findings were described in 292 patients, MRI findings in 231, and EUS findings in 115) [9], and the typical imaging findings were second duodenal portion wall thickening (88.8% of cases), which is usually eccentric (81.8%), associated with the presence of duodenal wall cysts (82.6%) and second duodenal portion increased wall enhancement (76.3%) [9]. The authors of the same review emphasized the role of CT as the first-line imaging modality, MRI as the second-level imaging modality of choice (in the case of inconclusive CT findings), and EUS as a problem-solving technique in difficult cases [9]. EUS has higher accuracy than CT and MRI in depicting duodenal wall changes and offers the possibility of obtaining cyto-histological samples but is limited by the invasiveness and lower tolerance [9]. In contrast to typical patients with paraduodenal pancreatitis, our patient is female and has never over-consumed alcohol.

Available data on the relationship between AIP and PDAC are scarce. Several studies have investigated the risk of various malignancies in patients with IgG4-RD, with contradictory results. Hirano et al. analyzed one hundred and thirteen patients with IgG4-RD (among them ninety-five with AIP) in Japan, for whom malignancy was not diagnosed at the time of IgG4-RD onset and the follow-up period was longer than six months, finding 15 malignancies (pancreatic cancer in two patients) [10]. The incidence of total malignancies in the same study was similar to that observed in the general population [10]. Patients (*n* = 138) with AIP type 1 and more than two years of follow-up were included in the study from South Korea, and no pancreatic cancer occurred during the follow-up [11]. In a multicenter, retrospective cohort study from Japan, 108 patients who met the Asian diagnostic criteria for AIP were included, and 18 cancers were found (stomach, lung, non-Hodgkin lymphoma, prostate, colon, bile duct, and thyroid) in 15 patients (13.9%) during the median follow-up period of 3.3 years [12]. The relative risk of cancer among AIP patients at the time of AIP diagnosis was 4.9 (95% CI 1.7–14.9), and the authors concluded that the patients with AIP are at a high risk of having various cancers, and the highest risk of cancer was in the first year after AIP diagnosis; interestingly, none of them developed pancreatic cancer [12]. Schneider et al. retrospectively analyzed data from twenty-eight patients with AIP from a German center and retrieved the expected cancer incidence for the general population from the German Cancer Registry, finding six malignant diseases (but no pancreatic cancer) in five patients with AIP [13]. The overall incidence rate of malignant diseases in AIP patients was significantly increased compared to the expected incidence in the German population [13]. One hundred and sixteen patients with AIP from a prospectively maintained database at the Mayo Clinic were compared with the control group from the primary care clinics, and after a median follow-up of over three years, the risk of developing cancer after the index date was similar in AIP and control subjects. (The three most commonly diagnosed malignancies in the AIP group were prostate cancer, lymphoma, and bladder cancer [14]). A scoping review focused on patients with AIP identified 33 cases of PDAC in patients with AIP (mostly AIP type 1), typically located in the part of the pancreas affected by AIP. (The synchronous occurrence of PDAC and AIP was reported in 33% of patients, and metachronous occurrence was reported in 67% [15].) In the metachronous group, the median period between diagnoses was 66.5 months, and the majority of cancers (86%) occurred more than two years after AIP diagnosis [15]. According to the European Guideline on IgG4-related digestive disease, life-long surveillance is recommend for patients with IgG4-RD; however, caution is needed since data on the potential association between IgG4-RD and cancer might be biased due to the more careful surveillance of patients with IgG4-RD and AIP [4]. In contrast to most patients with AIP type 1, our patient is female and has never had symptoms of any autoimmune diseases or other organ involvement.

IPMN of the pancreas is characterized by cystic dilatation of the main or branch pancreatic duct and is a precursor of pancreatic cancer [8]. A recently published retrospective study explored the relationship between AIP and IPMN, showing that the prevalence of IPMN in patients with AIP was higher than that observed in the general population (21% vs 8–10%), and high-risk stigmata were more frequently observed in IPMN occurring together with AIP compared to isolated IPMN (*p* < 0.05) [16].

## 5. Conclusions

The diagnosis of AIP and paraduodenal pancreatitis is not always straightforward, and in some cases, it is not easy to differentiate them from pancreatic cancer. Surgery should be considered in patients when a suspicion of malignant/premalignant lesions cannot be excluded after a complete diagnostic work-up.

## Data Availability

No new data were created or analyzed in this study. Data sharing is not applicable to this article.
